# Methodology Development for Investigating Pathophysiological [^18^F]-FDG Muscle Uptake in Patients with Rheumatic Musculoskeletal Diseases

**DOI:** 10.3390/biomedicines13020465

**Published:** 2025-02-14

**Authors:** Maia Sobejana, Mustafa Al Beiramani, Gerben J. C. Zwezerijnen, Anneke van der Kooi, Joost Raaphorst, Carel G. M. Meskers, Martin van der Esch, Conny J. van der Laken, Maarten M. Steinz

**Affiliations:** 1 Reade, Center for Rehabilitation and Rheumatology, 1056 AB Amsterdam, The Netherlands; 2 Department of Rheumatology and Clinical Immunology, Amsterdam University Medical Center, 1105 AZ Amsterdam, The Netherlands; 3 Department of Radiology & Nuclear Medicine, Amsterdam University Medical Center, VU, 1081 HV Amsterdam, The Netherlands; 4 Department of Neurology, Amsterdam University Medical Center, 1105 AZ Amsterdam, The Netherlands; 5 Department of Rehabilitation Medicine, Amsterdam University Medical Center, Amsterdam Movement Sciences, 1081 HV Amsterdam, The Netherlands; 6 Health Faculty, Amsterdam University of Applied Sciences, 1067 SM Amsterdam, The Netherlands; 7 Amsterdam Movement Sciences (AMS), 1081 BT Amsterdam, The Netherlands

**Keywords:** rheumatoid arthritis, osteoarthritis, myositis, PET, [^18^F]-FDG, glucose metabolism, skeletal muscle, RMD

## Abstract

**Objectives:** This retrospective study explored the qualitative and quantitative assessment of F18-fluordeoxyglucose ([^18^F]-FDG) positron emission tomography and computed tomography (PET/CT) scans to assess pathophysiological muscle glucose uptake in patients with a rheumatic musculoskeletal disease (RMD). [^18^F]-FDG PET/CT detects metabolic activity via glucose uptake in tissues. This study aimed to determine the feasibility of quantitative assessment of [^18^F]-FDG uptake in muscles across three different RMDs compared to controls. **Methods:** In this study we analysed whole-body [^18^F]-FDG PET/CT scans from patients with rheumatoid arthritis (RA; *n* = 11), osteoarthritis (OA; *n* = 10), and idiopathic inflammatory myositis (IIM; *n* = 10), and non-RMD controls (*n* = 11), focusing on muscle-tracer uptake in specific muscle groups. Qualitative assessment visually identified regions with high [^18^F]-FDG uptake, followed by quantitative assessment using two methods: fixed volume-of-interest (VOI) and hotspot VOI. In the fixed VOI method, a VOI was placed in the respective muscle at a fixed position (50% height from proximal to distal end) on PET/CT images. In the hotspot VOI method, the VOI was placed at the site of the highest [^18^F]-FDG uptake observed during qualitative assessment. Standardised uptake values (SUVs) were determined for different muscle groups between RMDs and controls. **Results:** Qualitative assessment revealed a heterogenous uptake pattern of [^18^F]-FDG that was found in 93% of quadriceps and hamstring muscles, while other muscles displayed either heterogenous or homogenous patterns. A Bland–Altman analysis showed that the hotspot VOI method had a higher sensitivity in detecting differential [^18^F]-FDG uptake in muscles. Across all muscle groups, patients with IIM had the highest [^18^F]-FDG uptake, followed by patients with OA and RA, respectively. **Conclusions:** [^18^F]-FDG PET/CT enables qualitative and quantitative differentiation of muscle glucose uptake in patients with RA, OA, and IIM, at both individual muscle and patient group levels. The hotspot method and SUV_peak_ are recommended for quantitative assessment. High [^18^F]-FDG uptake in multiple muscle groups suggests pathophysiological glucose metabolism in RMD-affected muscles.

## 1. Introduction

Skeletal muscle weakness and muscle fatigue (hereafter referred to as muscle weakness) are frequently experienced by patients with rheumatic musculoskeletal diseases (RMDs) [1,2,3,4]. It is a clinical problem (prevalence up to 60%), with a high socio-economic burden, decreasing patients’ ability to work, engage in social activities and maintain their quality of life [5,6]. RMDs are defined by the European Alliance of Associations for Rheumatology (EULAR) as “a diverse group of diseases that commonly affect the joints, but can affect any organ of the body”, which includes muscles [7]. In RMDs that comprise idiopathic inflammatory myopathies (IIM), the underlying mechanism of muscle weakness is known to be caused by primary muscle inflammation [8]. However, in RMDs that affect the joints, such as rheumatoid arthritis (RA) [1,9] and osteoarthritis (OA) [10,11], the underlying pathogenesis of muscle weakness is not clear, and there is a need for improved clinical quantitative assessments.

Muscle weakness is associated with altered muscle glucose metabolism [12,13,14]. Muscle glucose metabolism is an intricate process that involves aerobic and anaerobic breakdown to provide energy in the form of adenosine tris-phosphate (ATP) for muscle force production [14]. There is emerging evidence of altered muscle glucose metabolism in RMDs [15,16,17,18]. In RA, impaired muscle glucose metabolism has been linked to insulin resistance [15], altered glycolysis [19], and disruptions in oxidative phosphorylation [20]. While evidence of altered glucose metabolism in OA is limited, the systemic nature of metabolic dysregulation [21] and the onset of insulin resistance [22] in OA suggest potential parallels in muscle tissue. Similarly, in IIM, increased muscle glucose uptake has been associated with intramuscular inflammation [23,24] as well as altered glycolysis and oxidative phosphorylation [25]. To study pathophysiological muscle glucose metabolism throughout the body and at the cellular level without the need of a muscle biopsy, whole-body [^18^F]-FDG PET (nowadays usually combined with computed tomography (PET/CT)) is used [26].

Despite the fact that [^18^F]-FDG PET/CT is often used in clinical practice, pathophysiological [^18^F]-FDG uptake in specific muscles of RMD patients is rarely evaluated. It has been shown that increased [^18^F]-FDG muscle uptake correlates with muscle pathology in biopsies obtained from patients with IIM [27]. This illustrates the possible clinical implications of [^18^F]-FDG PET/CT imaging in RMDs to non-invasively demonstrate muscle pathology throughout the whole body without the need for an invasive biopsy. Correct determination of pathophysiological [^18^F]-FDG uptake is therefore essential. Both qualitative and quantitative methods can be used to assess [^18^F]-FDG muscle uptake in RMDs. While a qualitative assessment offers a visual and subjective interpretation, it may not always provide sufficient accuracy in identifying pathophysiological tracer uptake. Quantitative methods offer measurable and objective data, enhancing reliability. Overall, there is insufficient knowledge available about (1) the suitability and best applicable quantitative methodology for PET/CT in assessing [^18^F]-FDG uptake in muscles of patients with RMD [28] and (2) the variability in [^18^F]-FDG PET/CT muscle uptake between muscle groups in patients with RMD and controls.

Therefore, this is a methodological study with the aim of exploring which [^18^F]-FDG PET/CT imaging approach can be used to quantitatively assess pathophysiological [^18^F]-FDG PET/CT uptake in muscles across three different RMDs (patients with IIM, as a positive control, OA and RA) and in non-RMD controls (as negative control).

## 2. Materials and Methods

### 2.1. Study Approval and Informed Consent

[^18^F]-FDG PET/CT scans were collected from a previous study on patients with RA and OA conducted at Amsterdam UMC [29] and from patients with IIM and non-inflammatory control persons who had an [^18^F]-FDG PET scan for clinical purpose within the last 5 years and did not have a neuromuscular disorder. This retrospective study was approved by the medical ethics committee (METC) of the Amsterdam University Medical Centre (Amsterdam UMC), VUmc location, in Amsterdam, The Netherlands (METC #2021.0535). Written informed consent under this explicit permit was obtained from all patients participating in this retrospective study following the METC rules and regulations for acquiring informed consent.

### 2.2. Participants

The PET/CT scans of the RA participants were from patients with clinically active disease (i.e., disease activity score of 28 joints (DAS28) ≥ 4). DAS28 was determined using the erythrocyte sedimentation rate (i.e., DAS28-ESR). Both patients with RA and OA were previously recruited from outpatient clinics of the departments of rheumatology at Reade and Amsterdam UMC, VUmc location, in Amsterdam, the Netherlands, for the HUMIRA PET study [29]. Patients with RA had to fulfil the ACR 1987 or 2010 classification criteria [30,31]. OA was diagnosed according to the 1986 clinical ACR criteria [32] and confirmed through radiography. The PET/CT scans of the 10 patients with IIM were from individuals with active disease under the clinical care of the neuromuscular department at Amsterdam UMC. All patients with IIM were treatment-naive (in terms of immunosuppressor) at the time of the PET and PET/CT scans, which were used to diagnose the patients at Amsterdam UMC. The patients with IIM had either dermatomyositis (*n* = 4), anti-synthetase syndrome (*n* = 4), or overlap myositis (*n* = 2). The control group consisted of PET/CT scans (*n* = 8) from persons without any diagnosed muscular disease (i.e., lung pulmonary nodules; uveitis; follicular lymphoma; tonsil carcinoma; active infection of the lunges; and diffuse B-cell lymphoma). All patients and control PET/CT scans were randomly selected from the entire pool of identified available PET/CT scans, and further selection was based on the availability of complete data (i.e., PET and CT scans available in combination with clinical records of the parameters of interest for this study). Exclusion criteria for patients with RA and OA were previously defined [29] and included hypersensitivity to any substance used for [^18^F]-FDG PET scans, active tuberculosis, severe infections, pregnancy, heart failure (NYHA class III/IV), cancer, and limited life expectancy (<12 months). Other exclusion criteria for this study included an age < 18 years old.

### 2.3. Image Analysis

#### 2.3.1. PET/CT Imaging

The [^18^F]-FDG PET/CT scans were acquired as previously described and in accordance with international guidelines [33], using Gemini TF or Ingenuity TF (Philips Healthcare) PET/CT scanners at Amsterdam UMC, VUmc location. The registered weight and same-day glucose test results were acquired from the electronic medical dossier. Participants fasted for >6 h before the tracer injection. [^18^F]-FDG (7 MBq per patient weight (kg) per emission acquisition duration (min)) was injected intravenously with a syringe and an intravenous drip (IV drip) [33]. Afterwards, the IV drip was flushed with 20 mL of 0.9% sodium chloride, and the residual activity in the syringe and IV drip was measured to determine the net injected dose. This was followed by a 90-min rest period, of which 30 min full bed rest with no conversation allowed. A low-dose CT scan (120 kV, 35 mAs) was performed for localization and attenuation correction, followed by the whole-body PET scan (acquisition time: 2 min per bed position from head to groin and 1 min per bed position from groin to toes). PET scans were normalized and corrected for scatter, decay, and attenuation [33]. The CT scans were used to determine the anatomical positioning of the muscles. Therefore, the correct anatomical positioning of the VOIs (as described further) could be achieved (i.e., through interpolation of the PET and CT images—hereafter referred to as PET/CT scans).

Patients with type 2 diabetes mellitus were asked to continue their oral medication in order to maintain their blood sugar levels. They were also asked to discontinue use of metformin at the time of the procedure and for 48 h after the procedure. For patients with type 1 diabetes mellitus, [^18^F]-FDG was injected 4 h after the subcutaneous injection of rapid-acting insulin and 6 h after the subcutaneous injection of short-acting insulin following the EANM procedure guidelines [33].

All PET/CT scans were checked for quality and comparability by two independent nuclear physicists of the Department of Nuclear Medicine at Amsterdam UMC prior to qualitative assessment and quantitative analysis.

#### 2.3.2. Qualitative Assessment

The obtained data were interpreted and analysed both visually (qualitatively) and semi-quantitatively by two independent experienced researchers, as described below. All images were viewed using the ACCURATE v17112022 software [34], which showed multiplanar and 3D reconstructions of the PET and CT images for qualitative and semi-quantitative assessments. The ACCURATE v17112022 software is publicly available, has been validated by Prof. Dr. Ronald Boellaard of Amsterdam UMC, and has been used in previous studies [35,36,37]. The evaluated muscles included the deltoid muscle, biceps brachii muscle, triceps brachii muscle, psoas muscle (between the L5–L1 junction in the sagittal plane), quadriceps muscle, and hamstrings. Each PET scan was reviewed and qualitatively assessed for the degree of [^18^F]-FDG uptake by researchers blinded to patient characteristics. Regions with [^18^F]-FDG uptake greater than or equal to that of the mediastinal blood vessels were considered to have increased uptake [20]. This reference was chosen because it has previously been documented as a site of increased [^18^F]-FDG uptake and has been used as a positivity criterion [27,38]. Regions judged to be positive were scored as increased uptake (1), and regions judged to be negative were scored as showing no uptake (0). The tracer uptake pattern in muscles was scored as either heterogeneous (i.e., unifocal, heterogeneous-multifocal, or heterogeneous-diffuse) or homogenous and was documented per muscle for each patient scan.

#### 2.3.3. Quantitative Analysis

**Figure 1:**
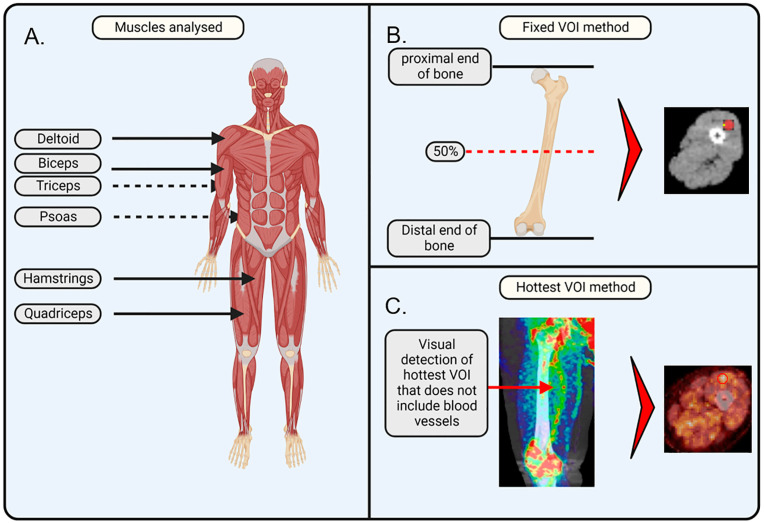
Schematic illustration of the applied methodology. (**A**) Muscles of interest that were analysed in this study are important for movement and posture and include the deltoid, biceps, triceps, psoas, quadriceps, and hamstrings. (**B**) Fixed volume of interest (VOI) method makes use of the anatomic position that is at 50% of the plane between the proximal and distal end of a bone. The VOI is drawn in the axial plane, ensuring it does not overlap with any veins or arteries. (**C**) Hottest VOI method makes use of the qualitative assessment of the muscle, where the VOI is drawn at the position of the most visually apparent [^18^F]-FDG uptake, with the consideration that the VOI should not overlap with any veins or arteries. The image was created with BioRender. Jansen, G. (2025) https://BioRender.com/w00w379 (accessed on 8 February 2025) and PET/CT images were added separately.

The quantified output measure of [^18^F]-FDG -PET/CT was the Standardised Uptake Value (SUV), which reflects [^18^F]-FDG uptake. SUV is defined as the activity concentration of the tracer in tissue/(injected activity/body size). To determine SUV, a volume of interest (VOI) was drawn over defined areas (see Figure 1). The peak SUV (SUV_peak_) was chosen as the outcome measure due to its ability to “maintain reproducibility of maximum SUV with improved statistics to reduce noise” [39]. The SUV for each VOI was corrected for body weight.

We applied and compared the following two methods of defined areas to assess SUVs of [^18^F]-FDG uptake.

Fixed VOI method. On the PET/CT images, VOIs were drawn on the PET/CT images at a fixed bone position relative to the muscle: VOIs for the biceps and triceps were drawn at the middle position (50% of the height from the proximal to the distal end) of the humerus; VOIs for the quadriceps and hamstrings were drawn at the middle position (50% of the height from the proximal to the distal end) of the femur; VOIs for the deltoids were drawn at the upper part (at the location of the deltoid) of the humerus; and VOIs for the psoas were drawn at the L5–S1 junction. The selected VOIs were transferred to the PET image to determine [^18^F]-FDG uptake, which was performed using the ACCURATE v06022022 software [34]. For the quadriceps and hamstrings, a VOI size of 20 mm^3^ was selected. For the deltoids, biceps, triceps, and psoas, a VOI size of 10 mm^3^ was selected. The VOI size was determined based on the muscle size to capture a representative and significant portion of the muscle while avoiding interference from other tissues, arteries, or non-muscle regions. The VOIs of deltoids, biceps, triceps, and psoas were 10 mm^3^, as these muscles were too small to encompass 20 mm^3^ inside their borders. A quality check on all VOI placements was performed by a nuclear physicist from the Nuclear Medicine department of Amsterdam UMC.

Hotspot VOI method. The VOI was drawn on the PET/CT image at the position where the qualitative assessment showed the highest visual [^18^F]-FDG uptake in the respective muscle. Using the ACCURATE v06022022 software, SUV values were extracted from the positioned VOIs. In brief, PET/CT scans were qualitatively evaluated in all planes (axial, sagittal, and coronal) for each respective muscle group. The plane that showed the highest [^18^F]-FDG uptake was selected. To avoid coincidence, two consecutive slices, prior or after the selected slice, had to exhibit similar increased [^18^F]-FDG uptake in the same area. Subsequently, a VOI was drawn on the selected plane at the spot that showed the highest uptake. The size of the VOI was identical to the sphere size used in the fixed VOI method (i.e., 20 mm^3^ for the quadriceps and hamstrings; 10 mm^3^ for the deltoids, biceps, triceps, and psoas).

To confirm the indication that [^18^F]-FDG uptake was symmetrical between the left and right muscles of the same group, SUV was extracted with the fixed VOI and hotspot VOI methods. A comparison was made between the left and right muscles of each muscle group (Supplementary Table S1).

### 2.4. Statistics

Data are presented as mean ± standard error of the mean (SEM). For all statistical analyses, normal data distribution was tested with the Shapiro–Wilk test, and equal variance was tested with the F test. First, to determine if there was a statistical difference between the left and the right muscle (irrespective of the RMD or control group/i.e., across all groups) in [^18^F]-FDG uptake, a paired T-test was used. Second, Pearson correlation was calculated to assess the relationship between the fixed and hotspot VOI and visualized using scatterplots. Subsequently, a Bland–Altman analysis was performed to determine whether there were systematic differences in the retrieved SUV values between the fixed or hotspot methods versus the mean of the two methods. Lastly, to compare the quantitative uptake of [^18^F]-FDG in the RMD groups and the controls, analysis of variance (ANOVA) was used, or the Kruskal–Wallis test was applied when the data were non-normally distributed or when equal variance could not be assumed. Post-hoc tests using Dunn’s test were performed to determine inter-group differences. All tests were performed for each muscle (deltoids, biceps brachii, triceps brachii, psoas, hamstrings, and quadriceps) separately and for the quantitative analysis. In all analyses, a *p*-value of less than 0.05 was considered statistically significant (* *p* < 0.05; ** *p* < 0.01; *** *p* < 0.001). All statistical analyses were performed using SPSS v. 26.0 or Prism 9 (GraphPad).

## 3. Results

### 3.1. Patient and Control Characteristics

A total of eleven RA (36.4% male), ten OA (20% male), ten IIM (30% male), and eight control PET/CT scans (50% male) were included in this study. The median age [interquartile range] of the patient groups were 60.9 [18.9], 60.1 [9.9], 52.0 [30.0], and 61.5 [34.8] years, respectively. The patient and disease characteristics are summarized in Table 1. Medication usage differed between patient groups; differences were mainly based on clinical diagnosis (i.e., 81.8% of the patients with RA and 90% of the patients with IIM received immunosuppressive medication (i.e., synthetic disease-modifying anti-rheumatic drugs (DMARDs), prednisone, and intravenous immunoglobulin treatment), while patients with OA did not. Antihypertensives were most used by patients with OA (60%), whereas prednisone was most used among patients with IIM (50%).

### 3.2. Qualitative Assessment of [^18^F]-FDG Muscle Uptake

**Figure 2:**
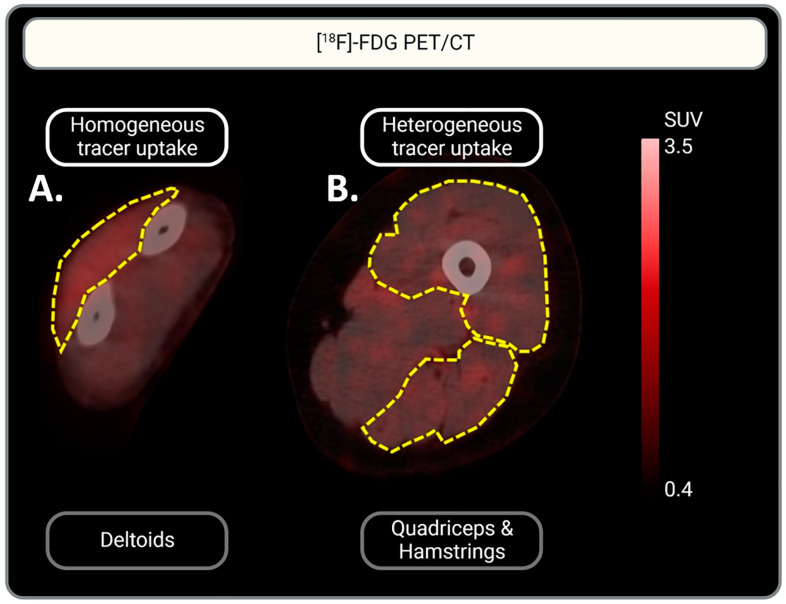
Representative illustration of homogeneous vs. heterogeneous [^18^F]-FDG muscle uptake. (**A**) Homogeneous [^18^F]-FDG uptake in the deltoid muscle is represented: a homogeneous red color can be observed. (**B**) Heterogeneous [^18^F]-FDG uptake in the quadriceps and hamstrings: multifocal red patches can be observed. Muscles are indicated by yellow dotted lines. Tracer uptake is represented in red. Scaling of [^18^F]-FDG uptake intensity is indicated by the scale-bar on the right: SUV is set between 0.4 and 3.5 for optimal visualization of tracer uptake.

One IIM scan did not include the left arm for the assessment of tracer uptake in the triceps and biceps. The qualitative assessment showed [^18^F]-FDG uptake in the muscles of all patient groups with various distribution patterns, homogenous vs. heterogenous (Figure 2). In 93% of the quadriceps and hamstring muscles, [^18^F]-FDG uptake was heterogenous, while uptake in other muscle groups showed both heterogenous and homogenous uptake. This accounted for all three RMDs: IIM, OA, and RA. In contrast, most tracer uptake in muscles (other than the quadriceps and hamstrings) of controls was homogenous. See Table 2 for a more detailed description of the uptake per muscle and per RMD group.

### 3.3. Quantitative Analysis of [^18^F]-FDG Muscle Uptake

To confirm that [^18^F]-FDG uptake was symmetrical between the left and right muscles of the same group, SUV was extracted with the fixed VOI and hotspot VOI methods, and a comparison was made between the left and right muscles of each muscle group (Supplementary Table S1). A significant difference in SUV was found in the psoas muscles when the fixed VOI method was used (mean ∆SUV left vs. right psoas = −0.091, *p* < 0.05). No significant differences were found in the uptake between sides for any of the other muscle groups with either the hotspot or fixed VOI method (Supplementary Table S1). Since the majority of muscle groups showed no significant differences between the left and the right muscles, the mean SUV of both left and right muscles for each muscle group per patient was used for further comparisons of the quantitative data.

### 3.4. Fixed vs. Hotspot VOI

**Figure 3:**
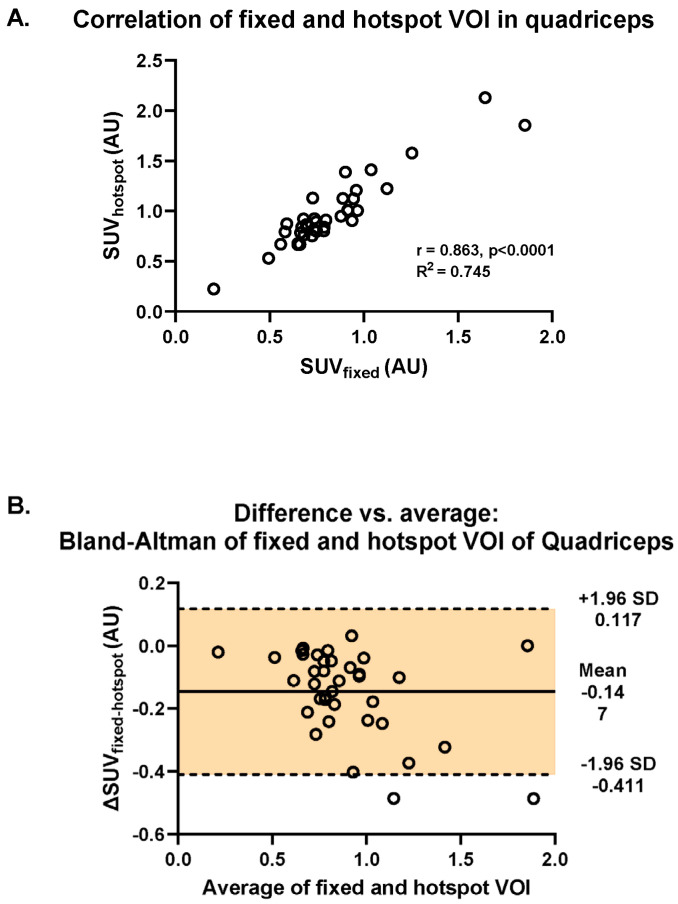
Investigation of the difference in intramuscular uptake of [^18^F]-FDG between the fixed and the hotspot methods in the quadriceps muscle across all RMDs and controls. (**A**) Visualization of the Spearman correlation between the fixed VOI and hotspot methods (R^2^ = 0.745). (**B**) Bland–Altman plot of the differences between fixed and hotspot VOI methods vs. the mean of the two methods.

There was a strong correlation between muscular [^18^F]-FDG uptake assessed with fixed VOI and hotspot methods (R^2^ = 0.865, *p* < 0.001; Figure 3A). In addition, using Bland–Altman analysis, we found that there was a systematic difference in SUVs between the two methods: most differences (ΔSUV_fixed-hotspot_) revealed negative values caused by higher SUVs retrieved by the application of the hotspot method (mean ΔSUV_fixed-hotspot_ = −0.147, 95%CI [−0.411, 0.117]) (Figure 3B). The difference of −0.147 is represented by the gap between the X axis, corresponding to a zero difference, and the parallel line to the X axis at −0.147. Furthermore, the spread of the difference around the mean was large (SD = +0.117, −0.411) (Figure 3B).

### 3.5. Comparison of Quantitative Uptake in the RMD Groups and Controls

**Figure 4:**
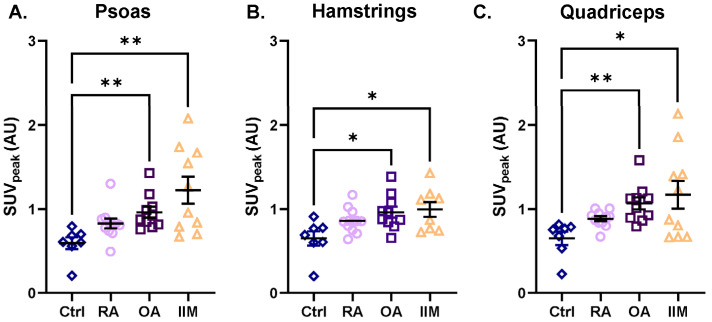
Intramuscular uptake of [^18^F]-FDG as assessed with the hotspot VOI in the psoas, hamstrings, and quadriceps of patients with RA, OA, and IIM in comparison to the controls. Quantification of [^18^F]-FDG uptake is represented by SUV_peak_ in (**A**) the psoas, (**B**) the hamstrings, and (**C**) the quadriceps for (from left to right) control persons, patients with RA, OA, and IIM. Quantitative data are presented as mean ± SEM, N = 7–11/group, * *p* < 0.05, ** *p* < 0.01, Kruskal–Wallis test with multiple comparison of the mean rank differences.

All muscle groups (i.e., deltoids, biceps brachii, triceps brachii, psoas, quadriceps, and hamstrings) showed the same trend of [^18^F]-FDG uptake among the RMDs when compared to the controls: the three RMD patient groups showed a trend of increased [^18^F]-FDG uptake (Figure 4, Supplementary Figure S1, and Supplementary Table S2). The uptake in the psoas, hamstrings, and quadriceps of patients with IIM was 2.2-, 1.5-, and 2.0-fold higher than in the controls, respectively (Figure 4A–C). In patients with OA, higher [^18^F]-FDG uptake was observed in all muscles compared to controls, which was significant in the psoas, hamstrings, and quadriceps (up to 1.6-fold higher) (Figure 4A–C). A slightly higher [^18^F]-FDG uptake (but not significant) was also observed in almost all muscles of patients with RA compared to controls, with the most pronounced difference in the biceps muscle (1.4-fold) (Figure 4A–C and Supplementary Figure S1B).

## 4. Discussion

The aim of this study was to explore the methodology of [^18^F]-FDG PET/CT imaging for the quantitative evaluation of pathophysiological [^18^F]-FDG uptake in muscles among individuals with three distinct rheumatic musculoskeletal disorder (RMDs), namely idiopathic inflammatory myopathies (IIM), osteoarthritis (OA), and rheumatoid arthritis (RA) as well as non-RMD controls. The findings indicate that [^18^F]-FDG PET/CT is a viable method for visualizing altered [^18^F]-FDG uptake in muscles across three different groups of RMDs. We carefully outlined the quantitative analysis approach to determine [^18^F]-FDG muscular uptake in RMDs to allow for the assessment and comparison of tracer uptake in different muscles and among different RMD patients. The average SUV_peak_ of the left and right muscles and the hotspot VOI method were used for the quantitative analysis. The results of this study provide insights that inform the recommended quantitative PET analysis methodology for a prospective [^18^F]-FDG PET/CT study to further investigate whether glucose metabolism in the muscles of different RMDs is altered and how the [^18^F]-FDG uptake patterns relate to muscle pathology.

The qualitative assessment revealed varying uptake patterns (homogenous and heterogenous) between different muscles in all RMD groups. Commonly, qualitatively defined homogenous [^18^F]-FDG muscle uptake patterns are expected in control muscles and are interpreted as physiological glucose uptake (linked to resting metabolism, exercise, and insulin-induced glucose use), while asymmetrical [^18^F]-FDG muscle uptake patterns indicate pathophysiological uptake [40]. This way of qualitatively defining pathophysiologic uptake may hold true for muscles of patients with RMD; however, this study showed that (i) not all muscles in healthy controls show a homogenous uptake pattern, and (ii) not all muscles affected by an RMD show a heterogenous uptake pattern. Our study thus demonstrates that a solely qualitative assessment may not be sufficient to identify pathophysiological [^18^F]-FDG muscle uptake in RMDs such as RA, OA, and IIM. The variability in [^18^F]-FDG muscle uptake per muscle type and patient group, as shown in the qualitative assessment (Table 1), highlights the additional value of a quantitative assessment.

In this study, we did not find any significant differences in almost all muscles on each side (left and right), and this statistical finding corresponded with the symmetrical uptake observed during the visual assessment. These findings are expected in patients with IIM, a disease characterized by symmetrical muscle weakness and symmetrical [^18^F]-FDG uptake, which has also been observed in other imaging studies in patients with poly- and dermatomyositis [24,27]. Interestingly, the uptake that we observed in the muscles of patients with OA was symmetrical, although it is generally believed to be an asymmetric disease [41]. Although it is generally accepted that OA is a disease of the joint, there is increasing evidence that molecular changes occur in the muscles surrounding the joints [11]. In general, a potential link between inflammation, molecular changes affecting [^18^F]-FDG uptake in the surrounding muscles, and muscle pathology in OA still needs further investigation. Regarding RA, there are currently no studies that have reported on the investigation of [^18^F]-FDG uptake in the muscles, despite the fact that [^18^F]-FDG-PET/CT has demonstrated intense and symmetrical uptake in (sub)clinically inflamed joints, both small and large joints [42]. Earlier reports suggest a systemic effect of chronic RA inflammation on skeletal muscles rather than a local effect driven by joint inflammation [1,43]. Similar to OA, the potential link between altered [^18^F]-FDG uptake, inflammation, and muscle pathology in RA needs to be further elucidated.

Two methods were compared for the quantification of [^18^F]-FDG muscle uptake: the fixed and hotspot VOI methods. Quantification of [^18^F]-FDG has certain drawbacks, including sensitivity to noise, scanner resolution, and tracer uptake time [44]. A first qualitative assessment followed by quantification of [^18^F]-FDG in a PET-positive region is a commonly applied technique in RMDs and oncological pathologies to circumvent false (positive/negative) interpretations of PET results [44,45]. When studying the effect of an RMD on skeletal muscle, i.e., the effect on [^18^F]-FDG uptake, the order of first conducting a qualitative assessment and subsequently performing a quantitative analysis is not evident. A strong linear correlation was found between the SUV values retrieved with the fixed VOI method and the hotspot VOI method. This would indicate that the choice of method would not change the outcome of the final comparison of [^18^F]-FDG muscle uptake between control persons and patients with RMD. However, the Bland–Altman analysis indicates a systemic bias between the two methods. In light of this result and possible heterogeneous uptake patterns, the choice of the fixed VOI method could potentially bias the retrieved SUVs and thus the subsequent comparison of [^18^F]-FDG muscle uptake between control persons and RMD patients. Therefore, we chose to use the hotspot method for further quantitative analysis (Figure 4 and Supplementary Figure S1). Moreover, if the uptake pattern had been homogeneous, the choice of the hotspot method would likely not change the retrieved SUV value. Another method that could have been applied is CT-based placement of a VOI over the entire muscle. This method has proven suitable for tissues where the size is small, such as the arterial wall, and a VOI can then easily be drawn that encompasses the entire region of interest [46]. However, such execution has not been proven for analyzing pathophysiological [^18^F]-FDG in skeletal muscles. Moreover, it would require a time-extensive determination of a VOI over the entire muscle, given the great size of muscles, i.e., the quadriceps and hamstrings. In oncology, artificial intelligence (AI)-derived models and automated segmentation software have been used for more accurate detection of [^18^F]-FDG uptake in an entire tumour [47,48]. The application of automatic segmentation may also be valuable for muscle analysis on PET/CT and should be investigated in future studies.

Due to the retrospective study design, only patients with an available PET/CT scan could be included. As a result, the analysis of [^18^F]-FDG muscle uptake in the RMD patients in this study is constrained in its ability to account for potential confounders. Control patients with diverse diagnoses, such as lymphoma and uveitis, are valuable comparators due to the lack of [^18^F]-FDG PET data in healthy subjects. Their conditions lack primary muscle inflammation, making them suitable for evaluating the quantitative methodology to distinguish between physiological and pathophysiological [^18^F]-FDG muscle uptake. However, it cannot be fully excluded that the pathologies of the controls may affect [^18^F]-FDG muscle uptake, potentially leading to altered uptake in comparison to healthy controls. Nevertheless, this study contributes to novel insights by the carefully outlined methodology for assessing [^18^F]-FDG muscle uptake in RMDs. This knowledge can be used in a prospective PET study investigating the relationship between pathophysiological [^18^F]-FDG muscle uptake and muscle pathology in RMDs.

## 5. Conclusions

[^18^F]-FDG PET enabled qualitative and quantitative assessments of muscle glucose uptake in RA, OA, and IIM patients at the individual patient level and at the muscle level. The quantitative hotspot method and SUV_peak_ PET/CT outcome measure offer a feasible approach that is recommended for assessing pathophysiological [^18^F]-FDG muscle uptake in RMDs. Assessing pathophysiological [^18^F]-FDG muscle uptake using the method described in this study can enhance the knowledge of muscle glucose metabolism in RA, OA, and IIM. This has the potential clinical implication that [^18^F]-FDG PET/CT could be used as a diagnostic tool for demonstrating muscle pathology in patients with RMDs. The results fuel the design of prospective clinical PET studies to investigate the relationship between pathophysiological [^18^F]-FDG muscle uptake and muscle pathology in these patient groups.

## Figures and Tables

**Table 1:** Baseline demographic, anthropometric, and disease-related characteristics of each group included in this study (*n* = 39).

	Ctrl (*n* = 8)	RA (*n* = 11)	OA (*n* = 10)	IIM (*n* = 10)
Demographic				
Age, median [IQR], years	61.5 [34.8]	60.9 [18.9]	60.1 [9.9]	52.0 [30.0]
Sex (M/F), n (% male)	4/4 (50%)	4/7 (36.4%)	2/8 (20%)	3/7 (30%)
Anthropometric				
Height, median [IQR], m	1.76 [0.11]	1.67 [0.13]	1.71 [0.09]	1.68 [0.3]
Weight, median [IQR], kg	69.0 [32.3]	66.0 [18.0]	83.5 [41.5]	78.7 [35.6]
BMI, median [IQR], kg·m^−2^	23.0 [8.0]	24.8 [5.2]	27.9 [13.3]	26.9 [7.0]
Disease Activity				
DAS28-ESR (0–10), mean (±SD)	-	5.1 (±0.6)	-	-
ESR, median [IQR], mm·h^−1^	-	16.0 [24]	6.0 [10]	57.0 [75]
CRP, median [IQR], mg·L^−1^	-	4.0 [8.0]	1.0 [2.0]	28.0 [38.9]
Disease duration, median [IQR], months	-	7.0 [18]	109.0 [94]	1.0 [55]
Diabetes mellitus, n (%)	0	3 (27.3%)	2 (20%)	0
Medication				
sDMARDs, n (%)		6 (54.5%)	0	1 (10%)
MTX only, n (%)		4 (36.4%)	-	2 (20%)
MTX + SSZ, n (%)		1 (9.1%)	-	-
MTX + HCQ, n (%)		1 (9.1%)	-	-
Analgesics, n (%)		6 (54.5%)	0	1 (10%)
Prednisone, n (%)		3 (27.3%)	0	5 (50%)
IVIG, n (%)		0	0	3 (30%)
Anti-hypertensives, n (%)		4 (36.4%)	6 (60%)	1 (10%)
Statins, n (%)		3 (27.3%)	3 (30%)	0

BMI, body mass index; ESR, erythrocyte sedimentation rate; CRP, C-reactive protein; DAS28-ESR, Disease Activity Score in 28 joints; sDMARDs, synthetic disease-modifying antirheumatic drugs; MTX, methotrexate; SSZ: Sulfasalazine; HCQ, Hydroxychloroquine; IVIG, intravenous immunoglobulin.

**Table 2:** Qualitative assessment of [^18^F]-FDG intramuscular per muscle (group).

	Control		RA		OA		IMM		Total	
Muscle/Muscle Group	Homo-Genous	Hetero-Genous	Homo-Genous	Hetero-Genous	Homo- Genous	Hetero-Genous	Homo-Genous	Hetero-Genous	Homo-Genous	Hetero-Genous
Quadriceps R	2	**6**	0	**11**	0	**10**	0	**10**	2	**37**
Quadriceps L	3	**5**	0	**11**	0	**10**	0	**10**	3	**36**
Hamstrings R	1	**7**	0	**11**	0	**10**	0	**10**	1	**38**
Hamstrings L	2	**6**	0	**11**	0	**10**	3	**7**	5	**34**
Triceps R	**7**	1	**6**	5	**5**	**5**	4	**6**	**22**	17
Triceps L	**6**	2	**9**	2	**6**	4	2	**7**	**23**	15
Biceps R	**7**	1	3	**8**	**5**	**5**	4	**6**	19	**20**
Biceps L	**7**	1	5	**6**	**7**	3	1	**8**	**20**	18
Psoas R	**7**	1	2	**9**	3	**7**	4	**6**	16	**23**
Psoas L	**7**	1	4	**7**	4	**6**	**5**	5	**20**	19
Deltoid R	**7**	1	2	**9**	2	**8**	3	**7**	14	**25**
Deltoid L	**6**	2	2	**9**	3	**7**	2	**8**	13	**26**

[^18^F]-FDG uptake was scored per left (L) and right (R) muscle (group) as either homogenous (0) or heterogenous (1). Bolded numbers represent the highest counts per muscle (group). One IMM PET scan did not include the left triceps and left biceps.

## Data Availability

All data relevant to this article are fully disclosed within the publication, and no supplementary data are available.

## Supplementary Materials

The following supporting information can be downloaded at: https://www.mdpi.com/article/10.3390/biomedicines13020465/s1, Figure S1. Intramuscular uptake of [^18^F]-FDG as assessed with the hotspot VOI in the deltoids, the biceps, and the triceps of patients with RA, OA, and IIM in comparison to controls. Quantification of [^18^F]-FDG uptake represented in SUV_peak_ in (A) the deltoids, (B) the biceps, (C) the triceps of the (from left to right) control persons and patients with RA, OA, and IIM. Quantitative data are presented as mean ± SEM, N = 7–11/group, * *p* < 0.05, ** *p* < 0.01, *** *p* < 0.001 Kruskal–Wallis test with multiple comparisons of the mean rank differences; Table S1. Comparison of differences in SUV_peak_ between fixed and hotspot methods for left vs. right across all muscle groups; Table S2. Statistical summary of [^18^F]-FDG uptake between the groups for the biceps, deltoids, and quadriceps derived from the peak SUV using the hotspot VOI method.
