# Supplementary material for: Methodology Development for Investigating Pathophysiological [18F]-FDG Muscle Uptake in Patients with Rheumatic Musculoskeletal Diseases
Source: Biomedicines. 2025 Feb 14;13(2):465. doi: 10.3390/biomedicines13020465 (PMC11853360; doi:10.3390/biomedicines13020465)
Supplement: Supplementary file 1 [file biomedicines-13-00465-s001.zip › biomedicines-3365879-supplementary.pdf]

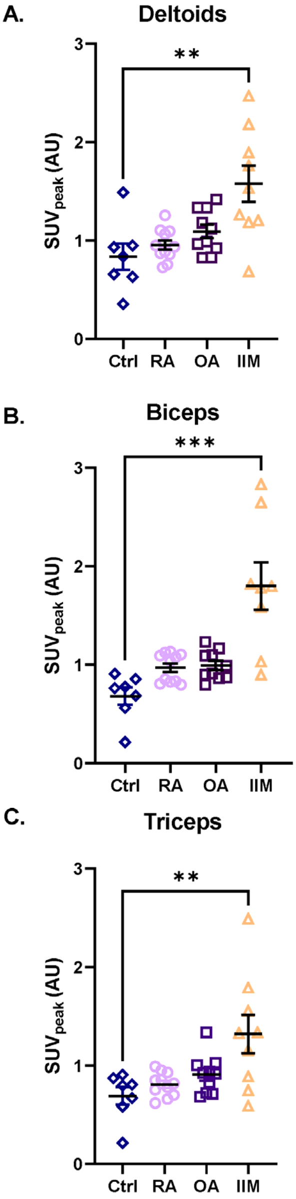

Figure S1. Intramuscular uptake of [^{18}F]-FDG, as assessed with the hotspot VOI method, in the deltoids, the biceps, and the triceps of patients with RA, OA, and IIM in comparison to the controls. Quantification of [^{18}F]-FDG uptake represented in SUV_{peak} in (**A**) the deltoids, (**B**) the biceps, (**C**) the triceps of the (from left to right) control persons and patients with RA, OA, and IIM. Quantitative data are presented as mean ± SEM, N = 7–11/group, * $p < 0.05$, ** $p < 0.01$, *** $p < 0.001$ Kruskal–Wallis test with multiple comparisons of the mean rank differences.

Table S1. Comparison of differences in peak SUV between, fixed and hotspot methods for left vs. right across all muscle groups.

	Fixed VOI		Hotspot VOI	
	**Δ Mean**	***p*-Value**	**Δ Mean**	***p*-Value**
Deltoids	−0.027	NS	−0.081	NS
Biceps brachii	0.003	NS	0.009	NS
Triceps brachii	0.014	NS	0.006	NS
Psoas	−0.091	0.002	−0.054	NS
Quadriceps	−0.003	NS	−0.078	NS
Hamstrings	−0.003	NS	−0.086	NS

* $p < 0.05$, NS: non-significant.

Table S2. Statistical summary of $[^{18}F]$-FDG uptake between the groups for the biceps, deltoids, and quadriceps derived from the peak SUV using the hotspot VOI method.

Muscle	Ctrl (N = 7)	RA (N = 11)	OA (N = 10)	IIM (N = 7)	*p*-Value
Psoas	0.59 (±0.07)	0.83 (±0.06)	0.96 (±0.07)	1.22 (±0.16)	0.0017 $^{+}$
Hamstrings	0.65 (±0.09)	0.86 (±0.05)	0.96 (±0.07)	0.99 (±0.09)	0.0126 *
Quadriceps	0.65 (±0.08)	0.88 (±0.03)	1.06 (±0.07)	1.17 (±0.17)	0.006 $^{+}$
Deltoids	0.84 (±0.13)	0.96 (±0.05)	1.09 (±0.07)	1.67 (±0.23)	0.004 $^{+}$
Biceps	0.68 (±0.09)	0.97 (±0.04)	0.99 (±0.05)	1.65 (±0.22)	<0.001 $^{+}$
Triceps	0.69 (±0.09)	0.81 (±0.04)	0.91 (±0.06)	1.22 (±0.19)	0.0023 *

Data = mean SUVpeak (±SEM), AU, * $p < 0.05$.
